# Impact of body composition on vitamin D requirements in healthy adults with vitamin D deficiency

**DOI:** 10.3389/fendo.2025.1421663

**Published:** 2025-07-03

**Authors:** Dexing Dai, Yali Ling, Feng Xu, Haibo Li, Rui Wang, Yingying Gu, Xuedi Xia, An Xiong, Ruoman Sun, Lei Qiu, Ya Ding, Yixin Yu, Xueyang Cai, Zhongjian Xie

**Affiliations:** National Clinical Research Center for Metabolic Diseases, Hunan Provincial Key Laboratory of Metabolic Bone Disease, and Department of Metabolism and Endocrinology, The Second Xiangya Hospital of Central South University, Changsha, Hunan, China

**Keywords:** vitamin D deficiency, vitamin D supplementation, vitamin D bioavailability, body fat, nutrition

## Abstract

**Background:**

Previous studies have shown that individuals with high body mass index typically require high doses of vitamin D supplementation to correct vitamin D deficiency. However, it is unclear which specific body composition is the determining factor affecting the bioavailability of vitamin D after supplementation. The aim of this study was to determine which body components affect the bioavailability of vitamin D.

**Methods:**

In order to ensure the compliance of the study subjects and avoid the impact of sunlight on vitamin D_3_ levels, the subjects received multiple intramuscular (i.m.) injections of vitamin D_2_ until their serum levels of 25-hydroxyvitamin D [25(OH)D] were above 30 ng/mL. All subjects received two i.m. injections of 600,000 IU vitamin D_2_, and dose adjustments were made every 6 weeks based on whether serum 25(OH)D levels were sufficient. The levels of serum 25(OH)D_2_ and 25(OH)D_3_ were determined by liquid chromatography tandem mass spectrometry. The body composition was measured using dual-energy X-ray absorptiometry and corrected using body fat mass index (FMI). Based on the 100% difference in 25(OH)D levels before and after vitamin D supplementation, the sample size was calculated, and 20 subjects would provide over 95% of the power to show the difference.

**Results:**

After two dose adjustment, the serum 25(OH)D levels of all subjects were above 30 ng/mL. The subjects were divided into ≤ 1,200,000 IU vitamin D_2_ (*n*=10) and ≥ 2,400,000 IU vitamin D_2_ (*n*=15) based on the i.m. dose of vitamin D_2_. The results showed that compared with subjects receiving ≤ 1,200,000 IU vitamin D_2_, subjects receiving ≥ 2,400,000 IU of vitamin D_2_ had a higher total body fat mass index (FMI), particularly with higher trunk fat content and high visceral adipose tissue mass. However, the dosage of vitamin D_2_ supplementation was not related to BMI and lean mass content.

**Conclusion:**

The body fat content, especially trunk fat content, is the main body component that affects the bioavailability of vitamin D in healthy adults. Healthy adults with high trunk fat content have low bioavailability of vitamin D and require relatively high dose of vitamin D to achieve sufficient levels.

**Clinical Trial Registration:**

http://www.chictr.org.cn, identifier ChiCTR2300070641.

## 1 Introduction

Vitamin D is an open-loop sterol fat soluble essential micronutrient relevant to numerous biological processes (1). It has been found that vitamin D is mainly stored in adipocytes through animal research using radio-labeled method (2). Vitamin D deficiency has become a global public health issue that may impair bone health and increase the incidence and progression of cancer, immune and cardiovascular diseases (3–6). Therefore, in order to maximize the protection of the health of bones and other organs, the nutritional status of human vitamin D needs to be sufficient. It is generally believed that serum levels of 25-hydroxyvitamin D [25(OH)D]< 20 ng/mL are vitamin D deficiency, and serum levels of 25(OH)D levels< 30 ng/mL are vitamin D insufficiency (7). The serum level of serum 25(OH)D is influenced by various factors including sunshine duration, ultraviolet radiation B (UVB) intensity, race, age, geographic latitude, dietary habits, dressing habits, and obesity. Previous studies have shown that serum levels of 25(OH)D in subjects with high normal body mass index (BMI) are lower than those in subjects with normal BMI, and serum levels of 25(OH)D in subjects with higher BMI have lower serum 25(OH)D levels than subjects with normal BMI after taking vitamin D_3_ orally (8–12). In addition, some cross-sectional studies have shown that increased waist circumference (WC) is related to the elevated risk of vitamin D deficiency in adults (13, 14). However, other cross-sectional studies have shown that WC and BMI are not associated with serum 25(OH)D levels (15, 16). The possible reason for the inconsistency in these results is that BMI or WC cannot reflect the amount of each body component, which may be a factor affecting the bioavailability of vitamin D. Other influencing factors include medication adherence and differences in sunlight exposure.

In the present study, in order to improve subject compliance and eliminate the influence of different sunlight exposure, we injected different doses of vitamin D_2_ into the muscles of subjects with different body components, and then observed whether the nutritional level of vitamin D reached the target level to determine which body component is the main factor affecting the bioavailability of vitamin D_2_.

## 2 Materials and methods

### 2.1 Study design

This 32-week, randomized, controlled clinical trial was conducted in healthy Chinese adults in Changsha, China, from March 2022 to October 2022, and lasted from early spring (March) to autumn (October). The aim of the study was to determine which body components affect the bioavailability of vitamin D. We chose intramuscular injection of vitamin D_2_ as the supplement method for vitamin D because the increased levels of 25(OH)D_2_ after vitamin D_2_ supplementation are not affected by sunlight. This study conforms to the principles outlined in the Declaration of Helsinki and has been approved by the Ethics Committee of the National Clinical Medical Research Center, the Second Xiangya Hospital, Central South University and registered in the China Clinical Trial Registration Center (ChiCTR number: ChiCTR2300070641). All participants signed informed consent forms before enrollment.

### 2.2 Participants

The study recruited adults with vitamin D deficiency [25(OH)D< 20 ng/mL] at the Second Xiangya Hospital. All participants understood the purpose of the trial and the benefits and possible risks during the study before enrollment. Subjects entered the screening process after signing the informed consent form, and subjects who met the enrollment criteria were numbered. The main enrollment criteria were based on the following terms: (1) serum 25(OH)D< 20 ng/mL; (2) aged between 18 and 60 years old; (3) No liver or kidney dysfunction or other serious diseases; (4) No long-term activity plan in tropical aeras within 6 months after the start of the experiment; (5) Voluntarily participate in the study and sign informed consent. The exclusion criteria were: (1) vitamin D supplements in the last 6 months; (2) a history of any drugs that affect vitamin D metabolism (such as phenytoin, phenobarbital, rifampicin) in the last 6 months; (3) unwillingness to participate in research; (4) a history of cognitive impairment or physical dysfunction that affects the completion of research; (5) Hypercalcemia, vitamin D poisoning, renal insufficiency, chronic diarrhea, hypoproteinemia or other diseases that affect vitamin D metabolism.

### 2.3 Sample size

Our previous studies have shown that a single i.m. injection of 600,000 IU vitamin D_2_ increases serum total 25(OH)D levels by 10.3 ng/mL (17). In the present study, the average baseline level of 25(OH)D was 7.6 ± 2.1 ng/mL. We estimated that subjects with vitamin D deficiency would need multiple i.m. injections of 600,000 IU vitamin D_2_ to achieve sufficient levels of vitamin D, i.e. serum total 25(OH)D ≥30 ng/mL. We assumed that the vitamin D sufficient rate before supplementation was 0% and the vitamin D sufficient rate after supplementation was 100%. The sample size of this study was calculated based on 5% of the inspection level (*α*), 95% of the inspection efficacy (1-*β*), and a relative difference of 100% when vitamin D levels reached sufficient levels before and after supplementation. The following formula was used to calculate the sample size required for this study (18).


$$N=\frac{{\left[Z_{1-\frac{\alpha }{2}}\sqrt{2\bar{p}\left(1-\bar{p}\right)}+Z_{\beta }\sqrt{p1\left(1-p1\right)+p2\left(1-p2\right)}\right]}^{2}}{{\left(p1-p2\right)}^{2}}$$

*N*: sample size; p 1: the incidence of vitamin D sufficient before supplement (0%); p 2: the incidence of vitamin D sufficient after supplement (100%); p = ( p 1+ p 2)/2; Z*_1-α/2_*: the standard deviation corresponding to the α level; Z*_β_*: is the standard deviation corresponding to the level of 1-*β*; α: 0.05 and 1-*β*: 0.95.

In the sample size calculation, we set a significance level of 0.05 and a withdrawal rate of 20%. The sample size of 20 subjects supplementing with vitamin D_2_ would provide over 95% power to show differences.

### 2.4 Randomization and intervention

**Figure 1:**
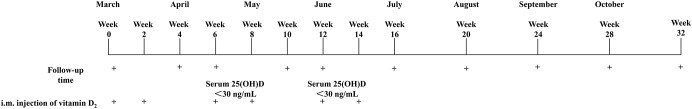
Follow-up and intervention procedures for the subjects. 25(OH)D, 25-Hydroxyvitamin D; i.m., Intramuscular.

Thirty volunteers were recruited and screened, among which subjects who met inclusion and exclusion criteria were enrolled and given the enrollment number in the order of arrival. Twenty-five subjects were received multiple i.m. injection of 600,000 IU vitamin D_2_ until the subject’s 25(OH)D level reached to the sufficient level. The follow-up and intervention procedures of the subjects are shown in **Figure 1**. Subjects were given a single i.m. injection of 600,000 IU vitamin D_2_ at the beginning of the study, followed by another 600,000 IU vitamin D_2_ after an interval of two weeks. The vitamin D_2_ supplement were adjusted every 6 weeks (week 6 and 12 of the study) based on whether serum 25(OH)D levels reached sufficient levels. Subjects whose serum 25(OH)D levels< 30 ng/mL continued to receive a single i.m. injection of 600,000 IU vitamin D_2_, followed by another 600,000 IU vitamin D_2_ after an interval of two weeks (at week 8 or 12 of the study). Finally, according to the dose of vitamin D_2_ supplementation, subjects was divided into ≤ 1,200,000 IU vitamin D_2_ group and ≥ 2,400,000 IU vitamin D_2_ group.

The vitamin D_2_ (Futai, 200,000 IU/1 mL) was purchased from Jiangxi Gannan Haixin Pharmaceutical Co., Ltd. (Ganzhou, Jiangxi, China). The vitamin D_2_ content corresponding to the indicated amount was confirmed by using Waters 1525–1489 high performance liquid chromatography (Waters, Milford, MA). All subjects were instructed normal diet, not to take calcium and oral vitamin D supplements, and not to take medications that affect vitamin D metabolism during the study period. The blood sample were collected at baseline and 4, 6, 10, 12, 16, 20, 24, 28, and 32 weeks after i.m. injection of vitamin D_2_. The 24-hour urine sample were collected at baseline and 4, 12, 16, 20, and 24 weeks after i.m. injection of vitamin D_2_. Body composition was measured by dual-energy X-ray absorptiometry (DXA) at 32 weeks after i.m. injection of vitamin D_2_. None of the subjects was withdrawn.

### 2.5 Biochemical marker determination

The serum samples from subjects were collected and stored at -80°C until the last batch of samples were collected and sent for testing. The vitamin D status was determined by measuring serum 25(OH)D levels, and Endocrine Society recommends achieving a 25(OH)D of 30 ng/mL (19). Due to the inability to distinguish between serum 25(OH)D_2_ and serum 25(OH)D_3_ when measuring serum 25(OH)D using chemiluminescence detection, serum 25(OH)D_2_ and 25(OH)D_3_ were measured by liquid chromatography tandem mass spectrometry (LC-MS/MS) using SCIEX 4500 MD LC-MS/MS at King Med Clinical Laboratory (Changsha, Hunan, China). The detection limits for serum 25(OH)D_2_ and 25(OH)D_3_ were 2.2 ng/mL and 2.6 ng/mL, respectively, with the intra-batch coefficient of variation (CV) approximately 3.0%. When the serum 25(OH)D_2_ level of the subject was below the detection limit, the value was recorded as 0 ng/mL.

Levels of serum calcium and 24-hour urinary calcium (24 h-Uca) were measured by arsenazo method to assess whether hypercalcemia and hypercalciuria after i.m. injection of vitamin D_2_, with an intra-batch CV was less than 3.5%, and an inter-batch CV was less than 6.0%. Hypercalcemia was defined as a fasting serum calcium level above 2.75 mmol/L (11 mg/dL). To calculate the urinary calcium/creatinine ratio, 24 h-Uca and 24-hour urinary creatinine (24 h-UCr) were measured. Both serum creatine and 24 h-UCr were detected using enzymatic methods, with an intra-batch CV was less than 5.0%, and an inter-batch CV was less than 5.0%. Hypercalciuria was defined as 24 h-Uca/24 h-UCr values above 0.3 mg/day or 24 h-Uca in females exceeds 6.25 mmol/day or in males exceeds 7.50 mmol/day (20).

Serum osteocalcin, intact parathyroid hormone (iPTH), bone formation markers including N-terminal propeptide of type I procollagen (P1NP), and bone resorption markers β C-terminal telopeptide of type I collagen (β-CTX) were measured by electrochemiluminescence in the Clinical Laboratory of Endocrinology at the Second Xiangya Hospital of Central South University (Changsha, Hunan, China). The intra-batch CV of osteocalcin, iPTH, P1NP and β-CTX were 1.8%, 2.7%, 3.0% and 3.5%, respectively, and the in inter-batch CV was 3.3%, 6.5%, 3.0%, 8.4%, respectively.

### 2.6 Body composition determination

Height (cm) and weight (kg) of the subjects were measured and their gender and age were recorded. The total body fat mass, total body lean mass, trunk fat mass, limb fat mass and visceral adipose tissue (VAT) mass were measured by DXA (Discovery, WiS/N87556, Hologic Inc., Bedford, MA, USA). Fat mass index (FMI) was calculated as total body fat mass (kg)/height^2^ (m^2^). Lean mass index (LMI) was calculated as total body lean mass (kg)/height^2^ (m^2^). Fat mass ratio (FMR) includes two indices, trunk fat mass (kg)/leg fat mass (kg) and trunk fat mass (kg)/limb fat mass (kg), respectively (21).

### 2.7 Statistical analysis

Statistical analysis was performed using SPSS software (SPSS Inc., Chicago, IL, version 26.0). All descriptive statistics are shown as mean ± standard (SD). Differences among different groups were determined by analysis of variance (ANOVA) for parametric data. Repeated measurements analysis was performed, and then multivariate-least significant difference *post hoc* tests were performed to analyze the differences among groups at the followed-up time point. Assessment of the incidence of hypercalcemia and hypercalciuria between groups using chi square test or Fisher’s exact probability method (theoretical frequency< 1). If *p* value less than 0.05, the differences were considered to be statistically significant.

## 3 Results

**Figure 2:**
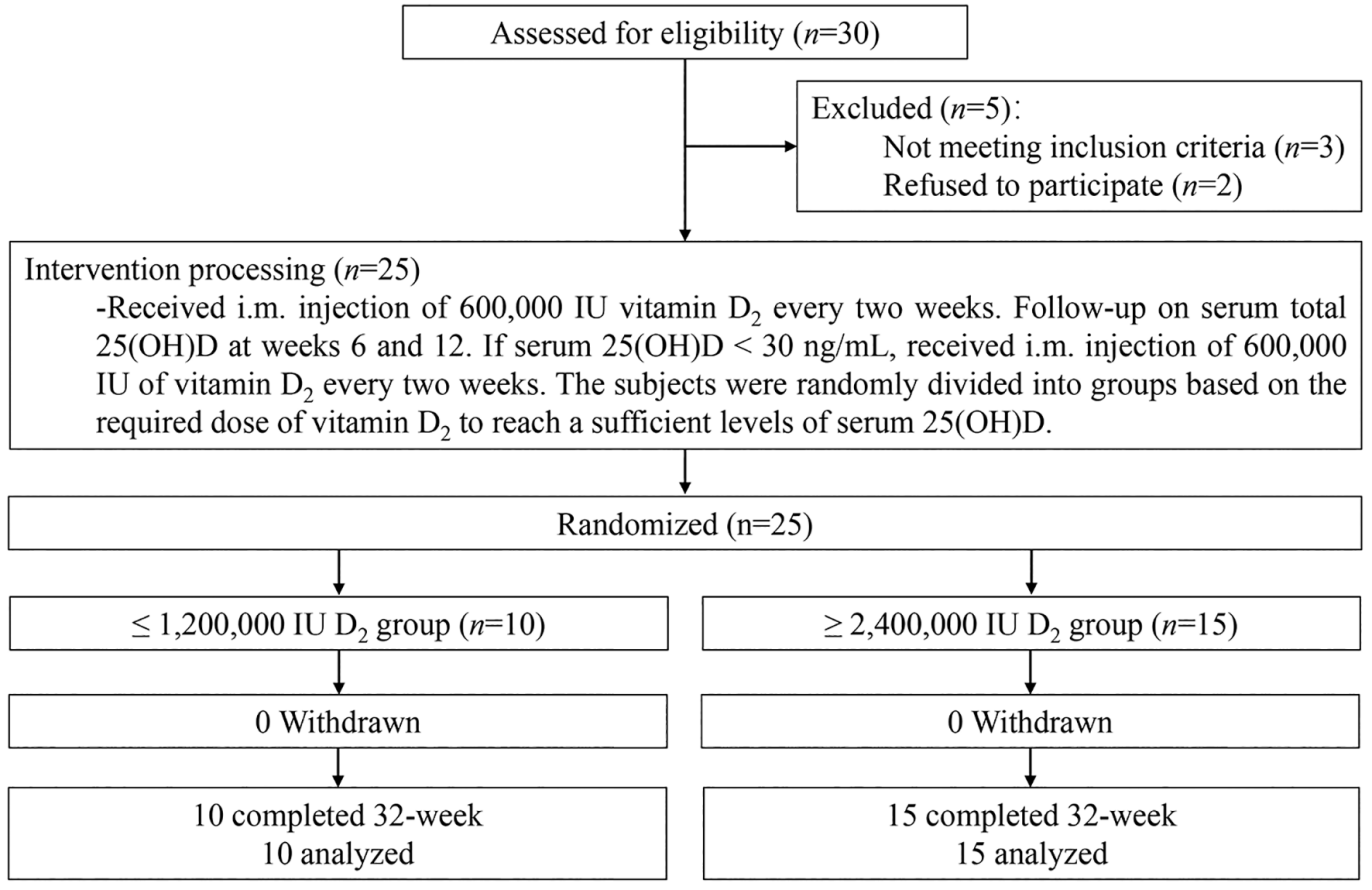
Flow diagram of the study. 25(OH)D, 25-Hydroxyvitamin D; i.m., Intramuscular; IU, International units.

Of the 25 participants enrolled at baseline, 25 finished the overall program and thus were included for further analysis (**Figure 2**). The characteristics of the subjects are presented in **Table 1**. Of the 25 subjects, 15 subjects (60.0%) were females and 10 subjects (40.0%) were males, and all subjects were vitamin D deficient. The mean age of the subjects was 26.3 ± 2.7 years, the mean height was 163.9 ± 10.4 cm, the mean weight was 61.4 ± 17.1 kg, and the mean BMI was 21.9 ± 3.4 kg/m^2^. The average baseline of total serum 25(OH)D levels of all subjects was 7.6 ± 2.1 ng/mL, and the serum 25(OH)D_2_ levels of all subjects were undetectable. According to the dose of vitamin D_2_ required for vitamin D to reach the sufficient status, the subjects were divided into divided into ≤ 1,200,000 IU vitamin D_2_ group (*n*=10) and ≥ 2,400,000 IU vitamin D_2_ group (*n*=15). There were no significant differences in age, height, weight, BMI, 25(OH)D, iPTH, β-CTX, P1NP, osteocalcin, serum calcium, serum creatinine, 24 h-Uca urinary calcium, and 24 h-Ucr levels between two groups (**Table 1**).

**Table 1:** Baseline characteristics of the subjects.

Characteristic	ALL (*n*=25)	≤ 1,200,000 IU D_2_(*n*=10)	≥ 2,400,000 IU D_2_ (n=15)	*p* value
Age(years)	26.3 ± 2.7	25.5 ± 2.0	26.9 ± 3.0	0.196
Height(cm)	163.9 ± 10.3	161.8 ± 8.9	165.3 ± 11.2	0.349
Weight(kg)	61.4 ± 17.1	55.2 ± 7.2	65.6 ± 20.5	0.003*
Females, *n*(%)	15 (60.0)	6 (60.0)	9 (60.0)	–
Males, *n*(%)	10 (40.0)	4 (40.0)	6 (40.0)	–
BMI(kg/m^2^)	21.9 ± 3.4	21.1 ± 2.6	23.5 ± 4.5	0.106
25(OH)D_2_(ng/mL)	0 ± 0	0 ± 0	0 ± 0	–
25(OH)D_3_(ng/mL)	7.6 ± 2.1	8.1 ± 1.6	7.3 ± 2.3	0.168
25(OH)D(ng/mL)	7.6 ± 2.1	8.1 ± 1.6	7.3 ± 2.3	0.168
iPTH(pmol/L)	6.9 ± 2.9	5.8 ± 2.0	7.7 ± 3.2	0.110
β-CTX(pg/mL)	409.8 ± 111.1	427.4 ± 105.3	398.1 ± 116.9	0.564
P1NP(ng/mL)	64.8 ± 23.0	73.2 ± 29.2	61.0 ± 17.1	0.372
Osteocalcin(ng/mL)	20.3 ± 9.1	23.3 ± 11.7	18.3 ± 6.5	0.303
Serum calcium(mmol/L)	2.38 ± 0.15	2.41 ± 0.17	2.37 ± 0.13	0.309
Serum creatinine(μmol/L)	64.2 ± 15.5	66.4 ± 12.4	62.8 ± 17.6	0.108
24 h-Uca(mmol/d)	3.95 ± 3.11	4.99 ± 2.83	3.26 ± 3.19	0.834
24 h-Ucr(mmol/d)	15.4 ± 10.8	16.2 ± 10.1	14.8 ± 11.5	0.442

BMI, Body mass index; 25(OH)D_2_, 25-Hydroxyvitamin D_2_; 25(OH)D_3_, 25-Hydroxyvitamin D_3_; 25(OH)D, 25-Hydroxyvitamin D; iPTH, Intact parathyroid hormone; β-CTX, β-C-terminal telopeptide of type I collagen; P1NP, N-terminal propeptide of type I procollagen; 24 h-Uca, 24-Hour urine calcium; 24 h-Ucr, 24-Hour urine creatinine; IU, International unit. *p, Significant difference between the ≤ 1,200,000 IU vitamin D_2_ group and the ≥ 2,400,000 IU vitamin D_2_ group.

**Figure 3:**
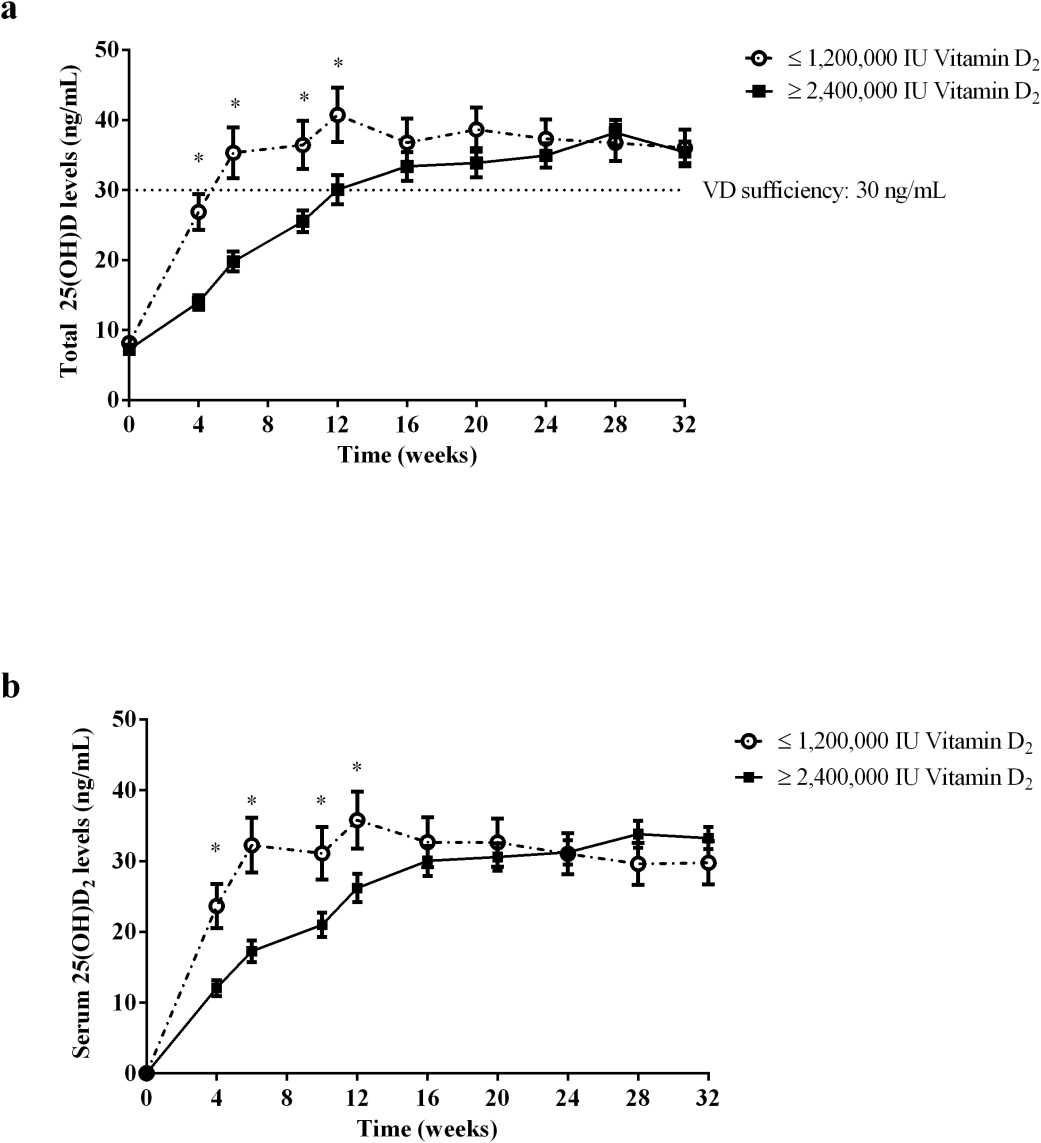
The total serum 25(OH)D **(a)** and 25(OH)D_2_ **(b)** level change over time after i.m. injection of vitamin D_2_. The subjects were divided into ≤ 1,200,000 IU vitamin D_2_ group (*n*=10) and ≥ 2,400,000 IU vitamin D_2_ group (*n*=15) according to the dose of vitamin D_2_ required reach sufficient vitamin D status. The dotted line shows the critical value of 30 ng/ml for vitamin D to reach the sufficient state. Data are shown as mean ± SD at each follow-up point. *P, significant difference between the ≤ 1,200,000 IU vitamin D_2_ group and the ≥ 2,400,000 IU vitamin D_2_ group at each follow-up time point. 25(OH)D, 25-hydroxyvitamin D; 25(OH)D_2_, 25-hydroxyvitamin D_2_.

**Figure 3** shows the levels of serum total 25(OH)D (**Figure 3a**) and 25(OH)D_2_ (**Figure 3b**) levels changes over time after multiple i.m. injections of vitamin D_2_. After i.m. injection of vitamin D_2_, the serum total 25(OH)D levels of subjects in two group increased significantly after the 4^th^ week. The total serum 25(OH)D levels of subjects with i.m. injection of ≤ 1,200,000 IU vitamin D_2_ group reached vitamin D sufficient status after 6^th^ week and remained stable until the end of study, with average peak value of 40.7 ± 12.3 ng/mL. However, the total serum 25(OH)D level of the subjects who received ≥ 2,400,000 IU vitamin D_2_ i.m. injection reached vitamin D sufficient status and remained stable at the 16^th^ week, with average peak value of 38.2 ± 7.1 ng/mL. In addition, at weeks 4, 6, 10, and 12, the serum total 25(OH)D levels in the ≤ 1,200,000 IU vitamin D_2_ group was significantly higher than those in the ≥ 2,400,000 IU vitamin D_2_ group (**Figure 3a**).

To eliminate the influence of sunlight, we used LS-MS/MS to determine the serum 25(OH)D_2_ levels at various follow-up points to analyze the bioavailability of vitamin D_2_ after intramuscular injection (**Figure 3b**). Serum 25(OH)D_2_ levels of subjects with i.m. injection of ≤ 1,200,000 IU vitamin D_2_ group and ≥ 2,400,000 IU vitamin D_2_ group reached vitamin D sufficient state after week 6 and week 16, respectively, and remained stable until the end of study. However, there was no significant difference in the average of serum 25(OH)D_2_ levels peak value between the ≤ 1,200,000 IU vitamin D_2_ group and the ≥ 2,400,000 IU vitamin D_2_ group (35.8 ± 12.8 ng/mL *vs.* 33.8 ± 7.4 ng/mL, *p* = 0.311). Similarly, at weeks 4, 6, 10, and 12, the serum 25(OH)D_2_ levels in the ≤ 1,200,000 IU vitamin D_2_ group was significantly higher than those in the ≥ 2,400,000 IU vitamin D_2_ group. Of note, the serum 25(OH)D_2_ levels of both groups were still above 30 ng/mL and remained stable at the end of study, probably due to the large amount of vitamin D stored in adipose tissue and slowly released into the bloodstream. These results suggest that subjects who received low-doses of vitamin D_2_ supplementation have a shorter time to reach the target level and have better bioavailability of vitamin D_2_.

**Figure 4:**
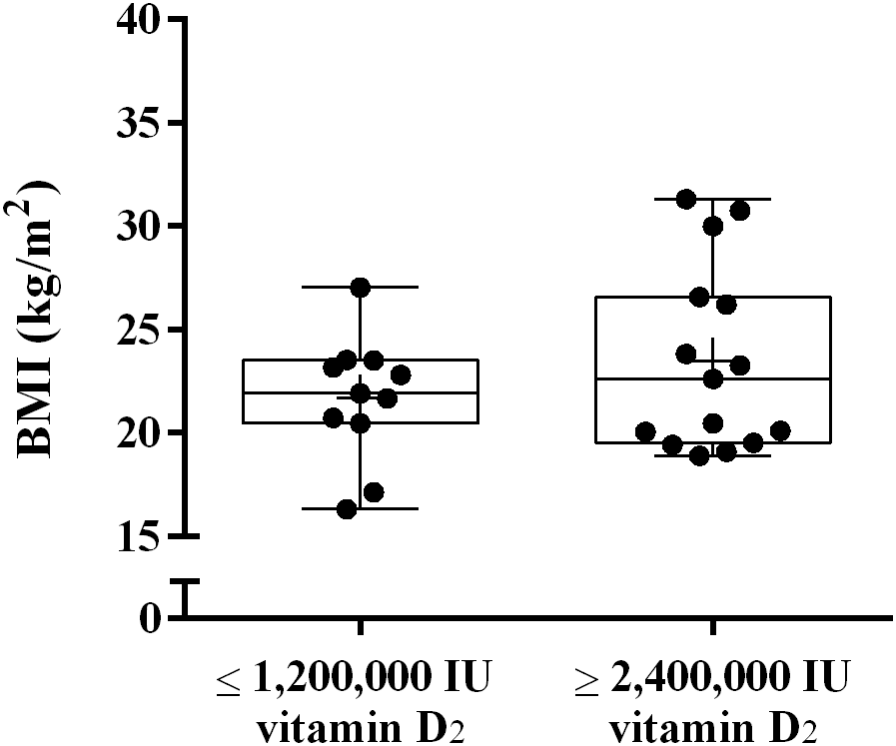
The difference in BMI between the ≤ 1,200,000 IU vitamin D_2_ group and the ≥ 2,400,000 IU vitamin D_2_ group. Data are shown as boxplots, including interquartile range, median, maximum and minimum value. BMI, Body mass index; IU, International units.

In the present study, we observed significant differences in the bioavailability of vitamin D among different subjects. To investigate which factors affect the bioavailability of vitamin D_2_, we analyzed whether there was any difference in BMI between the two groups. As shown in **Figure 4**, There was no significant difference in BMI between the ≤ 1,200,000 IU vitamin D_2_ group and the ≥ 2,400,000 IU vitamin D_2_ group (21.1 ± 2.6 kg/m^2^ *vs.* 23.5 ± 4.5 ng/mL, *p* = 0.106).

**Figure 5:**
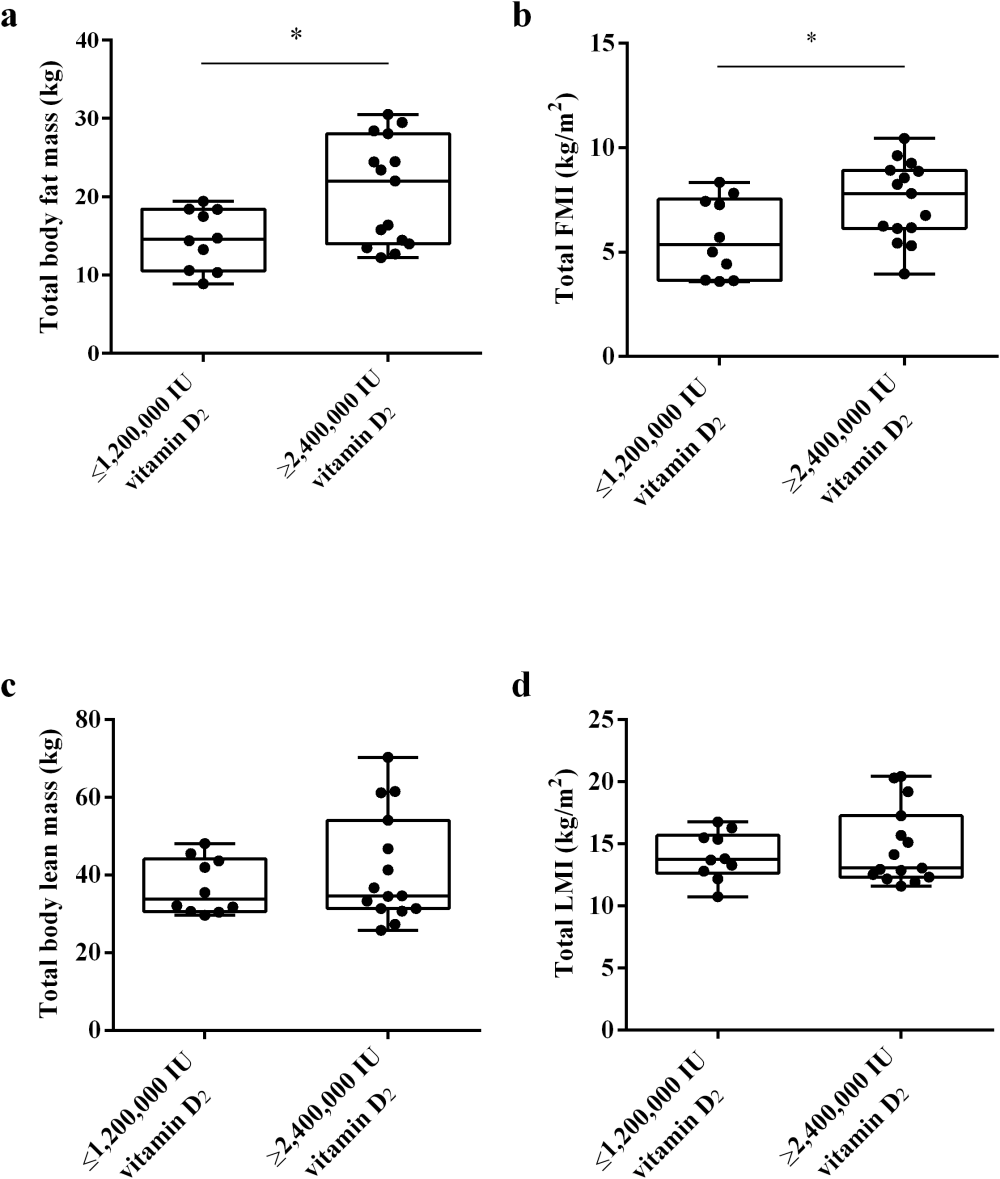
The difference in total body fat mass **(a)**, total FMI **(b)**, total body lean mass **(c)**, and total LMI **(d)** between the ≤ 1,200,000 IU vitamin D_2_ group and the ≥ 2,400,000 IU vitamin D_2_ group. Data are shown as boxplots, including interquartile range, median, maximum and minimum value. *P, significant difference between the ≤ 1,200,000 IU vitamin D_2_ group and the ≥ 2,400,000 IU vitamin D_2_ group. IU, International units; FMI, fat mass index; LMI, lean mass index.

Vitamin D is mainly stored in adipocytes, and the amount of adipose tissue may affect the level of serum 25(OH)D. To investigate whether the fat content affects the bioavailability of vitamin D_2_, we used DXA to measure the body components of subjects 32 weeks after intramuscular injection of vitamin D. As shown in **Figure 5**, the results showed that the total body fat mass and total FMI of subjects in ≥ 2,400,000 IU vitamin D_2_ group was significantly higher than those in the ≤ 1,200,000 IU vitamin D_2_ group (*p<* 0.05) (**Figures 5a, b**). These results suggest that subjects who require higher doses of vitamin D to achieve sufficient levels of vitamin D have higher total fat content.

In addition, we also analyzed whether muscle affects the bioavailability of vitamin D_2_, we used DXA to measure the lean mass and LMI of the subjects. Lean body mass refers to the muscle content. However, there was no statistically significant difference in total body lean mass and total LMI between two groups (**Figures 5c, d**). These results indicate that muscle content has no effect on the bioavailability of vitamin D_2_

**Figure 6:**
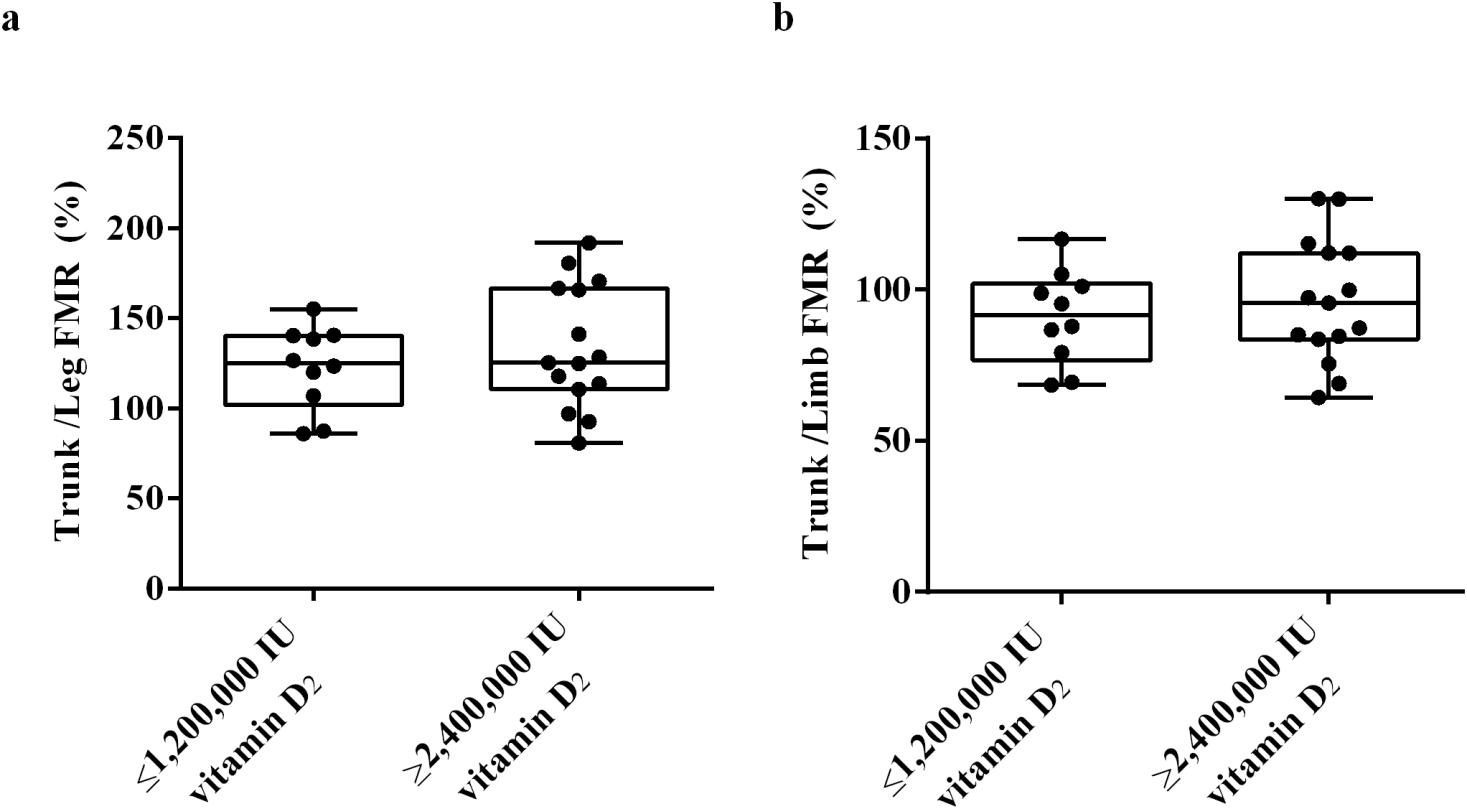
The difference trunk/Legs FMR **(a)**, trunk/limb FMR **(b)** between the ≤ 1,200,000 IU vitamin D_2_ group and the ≥ 2,400,000 IU vitamin D_2_ group. Data were shown as boxplots, including interquartile range, median, maximum and minimum value. IU, International units; FMR, Fat mass ratio.

**Figure 7:**
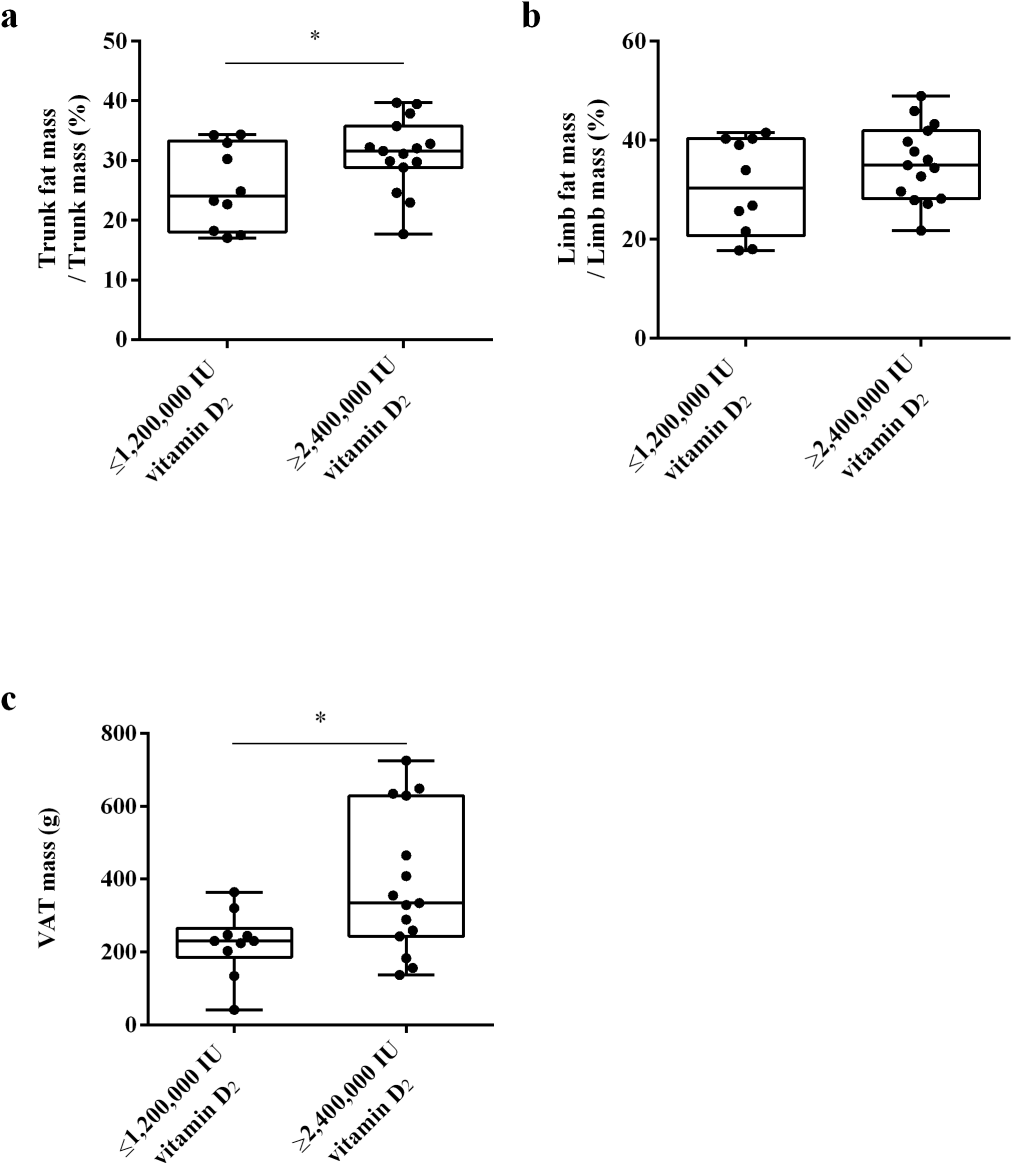
The difference in trunk fat mass/trunk mass **(a)**, limb fat mass/limb mass **(b)** and VAT mass **(c)** between the ≤ 1,200,000 IU vitamin D_2_ group and the ≥ 2,400,000 IU vitamin D_2_ group. Data are shown as boxplots, including interquartile range, median, maximum and minimum value. *P, significant difference between the ≤ 1,200,000 IU vitamin D_2_ group and the ≥ 2,400,000 IU vitamin D_2_ group. IU, International units; VAT, Visceral adipose tissue.

To clarify whether the differences in fat distribution are related to the required vitamin D dose to achieve sufficient vitamin D status, we used DXA to measure the trunk fat mass, limb fat mass and VAT mass of the subjects at week 32. The results showed that there was no significant difference in trunk/leg FMR and trunk/limb FMR between the ≥ 2,400,000 IU vitamin D_2_ group and the ≤ 1,200,000 IU vitamin D_2_ group. (**Figures 6a, b**). These results indicate that there was no significant difference in fat distribution between these two groups. Moreover, after trunk mass adjustment, the trunk fat content (%) of subjects in the ≥ 2,400,000 IU vitamin D_2_ group was significantly higher than those in the ≤ 1,200,000 IU vitamin D_2_ group (*p<* 0.05) (**Figure 7a**). However, after limb mass adjustment, there was no difference in the limb fat content (%) between the ≥ 2,400,000 IU vitamin D_2_ group and the ≤ 1,200,000 IU vitamin D_2_ group (*p* = 0.116) (**Figure 7b**). In addition, we analyzed the VAT in the trunk fat separately, and the results showed that the VAT mass of subjects in the ≥ 2,400,000 IU vitamin D_2_ group was significantly higher than those in the ≤ 1,200,000 IU vitamin D_2_ group (*p<* 0.05) (**Figure 7c**). Therefore, these results indicate that individuals with higher trunk fat content require a higher doses of vitamin D supplementation to achieve sufficient levels of 25(OH)D.

To determine whether multiple i.m. injections of vitamin D_2_ increase the risk of hypercalcemia and hypercalciuria, we measured levels of serum calcium and 24 h-Uca at weeks 0, 4, 12, 16, 20 and 24. There were no significant differences in serum calcium and 24 h-Uca between subjects receiving different doses of vitamin D_2_ at each follow-up time point (data not shown).

## 4 Discussion

In the present study, we regularly injected vitamin D_2_ into the muscles of the subjects, which not only ensured compliance but also ensured that the increase in 25(OH)D in the body was not affected by sunlight. We also used DXA to detect the body composition and demonstrated that body fat content has a greater impact on vitamin D_2_ bioavailability, and people with higher trunk fat content need to supplement higher doses of vitamin D to achieve adequate levels of vitamin D. However, the muscle content or BMI does not affect the bioavailability of vitamin D_2_. To our knowledge, this is the first clinical intervention study to investigate the effect of body fat content on the dosage required to achieve sufficient levels of vitamin D.

Previous animal studies have shown that vitamin D is mainly stored in adipocytes (2). In our study, we found there was no significant difference in BMI between different dosage vitamin D_2_ (≤ 1,200,000 IU of vitamin D_2_ group *vs.* ≥ 2,400,000 IU vitamin D_2_ group) required to achieve sufficient levels of 25(OH)D. AlQuaiz et al. found no significant correlation between serum 25(OH)D levels and WC in males and females after conducting multivariate analyses (15). Studies by Han et al. showed that after gender adjustment, BMI and WC were not associated with serum 25(OH)D (16). Wortsman et al. compared the difference in serum 25(OH)D_2_ or serum 25(OH)D_3_ levels between high BMI and normal BMI individuals who received oral vitamin D_2_ or whole body irradiation, and found that there was no significant difference in the peak of serum 25(OH)D_2_ or serum 25(OH)D_3_ levels between subjects with high BMI and subjects with normal BMI (22). In addition, in Wortsman’s study, the increase in serum 25(OH)D concentration in high BMI subjects was 57% lower than that in normal BMI subjects receiving equivalent ultraviolet radiation, but there was no difference in vitamin D_3_ precursor levels. The authors concluded that obesity-associated vitamin D insufficiency is likely due to the decreased bioavailability of vitamin D_3_ from cutaneous and dietary sources because of its deposition in body fat compartments (22). However, the results of some studies do not support this conclusion. Some clinical studies have demonstrated that serum 25(OH)D levels in subjects with high normal BMI are lower than those in subjects with normal BMI, and high weight subjects have lower serum 25(OH)D levels than normal weight subjects after vitamin D supplementation (23–25). Studies by Mattia Bellan et al. have shown that up to 95 percent of high BMI (35 to 70 kg/m^2^) individuals suffer from vitamin D deficiency, and subjects with the higher BMI had lower serum 25(OH)D levels (26). In a retrospective study, Monache et al. found that overweight patients had lower average 25(OH)D levels in winter and summer compared to normal weight individuals, usually in vitamin D deficiency status. Monache et al. also found that the higher BMI was significantly associated with lower serum total 25(OH)D levels, regardless of season or age (27). In some large-scale cross-sectional studies, the results showed a significant correlation between insufficient serum 25(OH)D levels and the risk of elevated WC (28, 29). These studies indicate that individuals with relatively high BMI or WC are prone to vitamin D deficiency. The reason for these inconsistent results may be related to the fact that WC and BMI cannot fully reflect body fat content. Therefore, it is necessary to clarify whether the body fat content will affect the bioavailability of vitamin D.

In the present study, we found that the total body fat mass and total FMI of subjects in ≥ 2,400,000 IU vitamin D_2_ group were significantly higher than those in the ≤ 1,200,000 IU vitamin D_2_ group (*p<* 0.05). However, there was no statistically significant difference in total body lean mass and total LMI between these two groups. These results indicate that for individuals with a high body fat content, supplementing with higher doses of vitamin D is necessary to achieve sufficient levels of 25(OH)D. Previous studies on the correlation between vitamin D supplementation dose and body fat content have not yielded consistent results. Gronborg et al. conducted a one-year randomized, double-blind, placebo-controlled trial and found no correlation between body fat content (fat mass/weight) and elevated levels of serum 25(OH)D elevation levels in the 400 IU/day vitamin D_3_ supplementation group (30). In Gronborg’s study, the reason for the negative results may be related to the low dose of vitamin D supplements received by the subjects. However, there was no significant correlation between the body fat content and elevated levels of 25(OH)D in the body of subjects after supplementing with 800–1000 IU/day of vitamin D_3_ (31, 32). In a randomized, double-blind, placebo-controlled study, 30 subjects received a single oral dose of 120,000 IU of vitamin D_3_, and the results showed that subjects with higher body fat content did not have lower serum 25(OH)D elevation levels (33). A randomized controlled study also showed that there was no significant correlation between changes in serum 25(OH)D levels and body fat mass in subjects receiving 400 IU/day vitamin D_3_ supplementation (n=21), but in subjects receiving 2000 IU/day vitamin D_3_ supplementation (n=23), individuals with high body fat mass showed a lower increase in serum 25(OH)D levels after vitamin D_3_ supplementation (n=23) (34). In another randomized controlled study, elderly subjects who received 500 mg calcium and 700 IU vitamin D_3_ daily after adjusting for baseline 25(OH)D levels, season and gender, showed a significant negative correlation between elevated levels of 25(OH)D and total body fat mass and central fat mass, but not peripheral fat mass (35). The reason for these inconsistent results may be the impact of sunlight on vitamin D_3_ levels, the difficulties in ensuring compliance with oral vitamin D supplements and the use of different measurement indicators.

Given the inconsistencies in prior findings, standardized adiposity assessment is critical for vitamin D research. To address this, we applied the FMI to adjust for differences in obesity caused by body size, which can directly reflect the fat mass component of body weight, and without interference from other components such as muscle mass (21, 36). Forsyth et al. measured FMI by measuring skinfold thicknesses in young and elderly individuals who received daily oral supplementation of 600 IU vitamin D_3_. The results showed that after adjusting for gender and region, baseline 25(OH)D levels in elderly subjects were significantly negatively correlated with fat mass and FMI, while there was no significant correlation in young subjects. In addition, the Forsyth et al. also found that after adjusting for baseline 25(OH)D levels, age, gender, and region, the increase in 25(OH)D levels was not significantly associated with fat mass and FMI (13). However, Forsyth et al. used skinfold thickness measurements to estimate FMI, rather than using DXA measurements to calculate FMI. Compared with skinfold thickness measurement, DXA measurement can accurately measure fat mass and calculate FMI. In the present study, there was no significant difference in FMI between males and females (6.32 ± 0.71 *vs.* 7.17 ± 0.49 kg/m^2^, *p* = 0.314). In addition, there was no significant difference in the dosage of vitamin D required for males and females to achieve sufficient levels of 25(OH)D. These results suggest that gender differences do not affect the dosage of vitamin D that needs to be supplemented.

While gender does not influence vitamin D dosage requirements, regional adipose tissue distribution may modulate its metabolic efficacy. A cross-sectional study has shown that 25(OH)D_3_ increases in abdominal subcutaneous fat tissue after supplementation with vitamin D_3_ (37). Another study showed that increases in 25(OH)D levels were correlated with decreases in the changes in all obesity indices, visceral, and abdominal adipose tissue during a year of weight loss through lifestyle changes (38). However, in these studies, limb fat content was not measured and it was unknown whether limb fat content was related to serum 25(OH)D levels. In the present study, the results showed that the trunk fat content (%) of subjects in the ≥ 2,400,000 IU vitamin D_2_ group was significantly higher than those in the ≤ 1,200,000 IU vitamin D_2_ group (*P*<0.05). However, there were no difference in limb fat content (%) adjusted by weight between the ≥ 2,400,000 IU vitamin D_2_ group and the ≤ 1,200,000 IU vitamin D_2_ group. Besides, the VAT content (%) of subjects in the ≥ 2,400,000 IU vitamin D_2_ group was significantly higher than those in the ≤ 1,200,000 IU vitamin D_2_ group. In addition, there was no statistically significant difference in trunk/leg FMR and trunk/limb FMR between two groups. Compared to limb fat deposits, trunk adipose tissue demonstrates a stronger inverse association with serum 25(OH)D levels. This differential relationship may be attributed to two primary mechanisms. First, the greater volumetric proportion of trunk fat relative to peripheral adiposity might magnify its metabolic influence on vitamin D homeostasis (39, 40). Second, compared to peripheral fat distribution, central adiposity exhibits enhanced secretion of proinflammatory cytokines that potentially suppress 25-hydroxylase enzymatic activity, thereby decreasing circulating 25(OH)D concentrations (41, 42).

In the present study, we also observed that the muscle content does not affect the bioavailability of vitamin D_2_. Previous studies also investigated this association and found similar results. Vitezova et al. found that lean body mass showed no association with vitamin D deficiency (43). Taken together, these findings suggest that body fat content rather than muscle content affects the bioavailability of vitamin D_2_.

In the present study, baseline serum 25(OH)D_2_ levels fell below detectable thresholds in the study cohort, a finding potentially attributable to the limited consumption of vitamin D_2_-rich foods (e.g., UV-exposed mushrooms) and insufficient use of vitamin D_2_ supplements among the Chinese population. Notably, serum 25(OH)D_3_ concentrations remained sufficient to maintain iPTH levels within normal physiological parameters, thereby preventing a clinically significant elevation of iPTH.

This study pioneers the application of body composition analysis to investigate how regional adipose tissue distribution influences vitamin D supplementation requirements. A key methodological strength lies in the standardized intramuscular vitamin D_2_ administration protocol, which ensured treatment compliance and eliminated confounding from sunlight-induced 25(OH)D fluctuations. Furthermore, we utilized DXA for precise body composition profiling and adopted FMI as a superior adiposity indicator to BMI. Limitations include the modest sample size, which may constrain the generalizability of findings, and the single-center design that may restrict population representativeness. Subsequent investigations should employ multi-center cohorts with demographically diverse populations to enhance external validity, complemented by stratified analyses of adipose-depot-specific vitamin D metabolism.

## 5 Conclusion

The body fat content, especially trunk fat content, is the main body component that affects the bioavailability of vitamin D in healthy adults. Healthy adults with high trunk fat content have low bioavailability of vitamin D and need to supplement relatively higher doses of vitamin D to achieve sufficient levels of vitamin D. However, the muscle content does not affect the bioavailability of vitamin D.

## Data Availability

The datasets presented in this article are not readily available because The data supporting the research results are not been publicly available, but reasonable requests can be made through the corresponding author. Requests to access the datasets should be directed to zhongjian.xie@csu.edu.cn.
